# Supplementary material for: Design, Synthesis and Functional Analysis of Cyclic Opioid Peptides with Dmt-Tic Pharmacophore
Source: Molecules. 2020 Sep 17;25(18):4260. doi: 10.3390/molecules25184260 (PMC7570497; doi:10.3390/molecules25184260)
Supplement: Supplementary file 1 [file molecules-25-04260-s001.pdf]

Supporting information

Design, Synthesis and Functional Analysis of Cyclic Opioid Peptides with Dmt-Tic Pharmacophore

Arijit Sarkar [1,2,†], Anna Adamska-Bartlomiejczyk [3,†], Justyna Piekielna-Ciesielska [3], Karol Wtorek [3], Alicja Kluczyk [4], Attila Borics [1,2] and Anna Janecka [3,*]

[1] Laboratory of Chemical Biology, Institute of Biochemistry, Biological Research Centre, Szeged, 62. Temesvári krt., H-6726 Szeged, Hungary; sarkar.arajit@brc.hu (A.S.); borics.attila@brc.hu (A.B.)
[2] Theoretical Medicine Doctoral School, Faculty of Medicine, University of Szeged, 97. Tisza L. krt., H-6722 Szeged, Hungary
[3] Department of Biomolecular Chemistry, Faculty of Medicine, Medical University of Lodz, Mazowiecka 6/8, 92-215 Lodz, Poland; anna.adamska-bartlomiejczyk@umed.lodz.pl (A.A.-B.); justyna.piekielna-ciesielska@umed.lodz.pl (J.P.-C.); karol.wtorek@umed.lodz.pl (K.W.)
[4] Faculty of Chemistry, University of Wroclaw, F. Joliot-Curie 14, 50-383 Wroclaw, Poland; alicja.kluczyk@chem.uni.wroc.pl
* Correspondence: anna.janecka@umed.lodz.pl; Tel.: +4842 272 57 06
† These authors contributed equally to this work.

Contents

Figure S1-S6. Analytical HPLC chromatograms of analogs **1-6** pS2

Table 1. Physicochemical data of analogs **1-6**.. pS4

Figure S6-S12. High resolution MS spectra of analogs **1-6** pS5

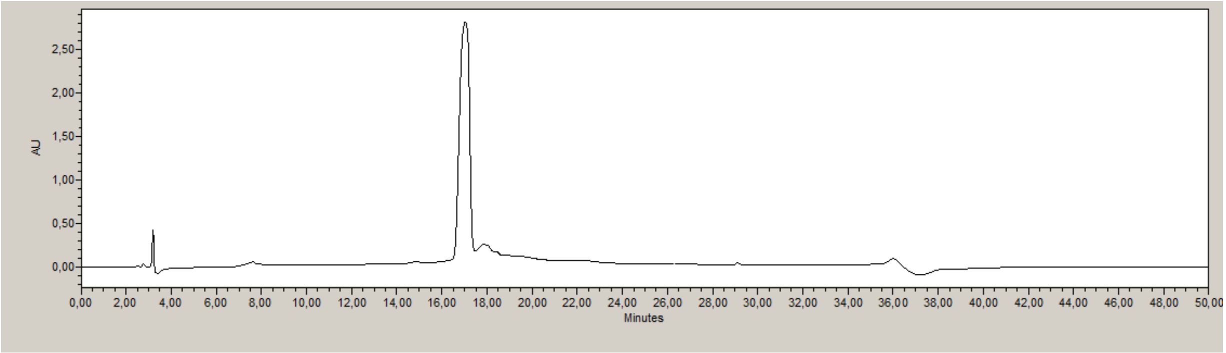

Figure S1. Analytical HPLC chromatogram of peptide Dmt-Tic-c[D-Lys-Phe-Phe-Asp]NH_2 (**1**).

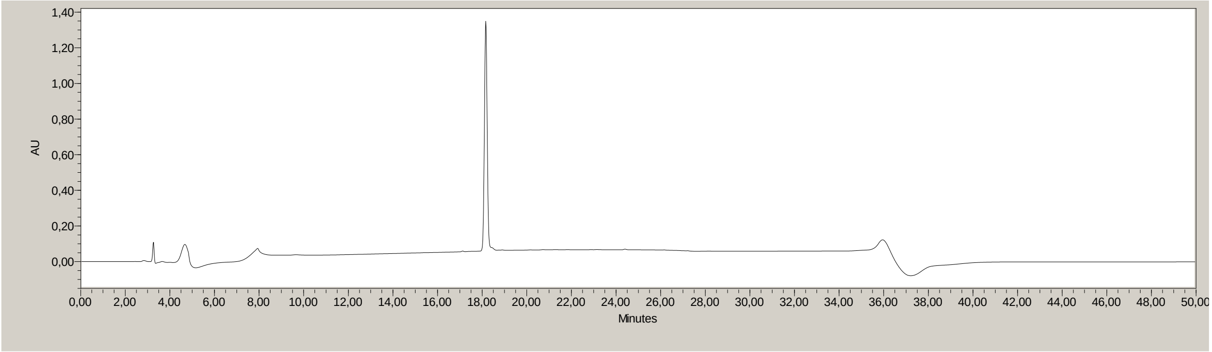

Figure S2. Analytical HPLC chromatogram of peptide Dmt-Tic-c[D-Lys-Phe-D-2Nal-Asp]NH_2 (**2**).

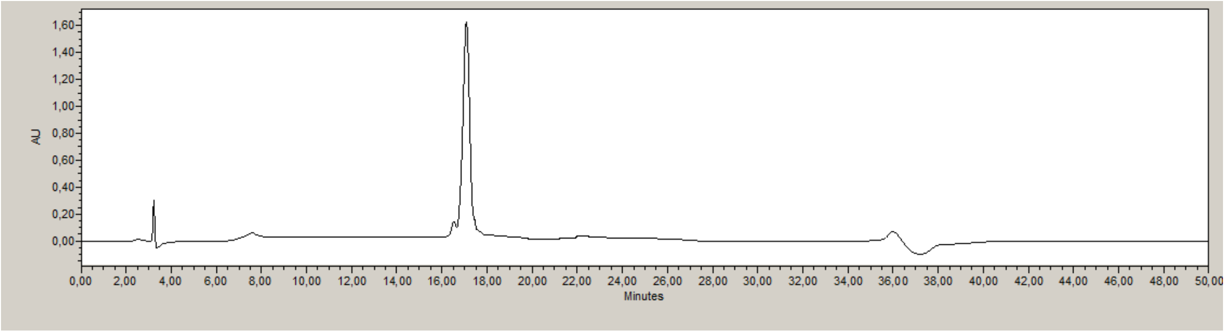

Figure S3. Analytical HPLC chromatogram of peptide Dmt-Tic-c[D-Lys-Phe-2,4F_2-Phe-Asp]NH_2 (**3**).

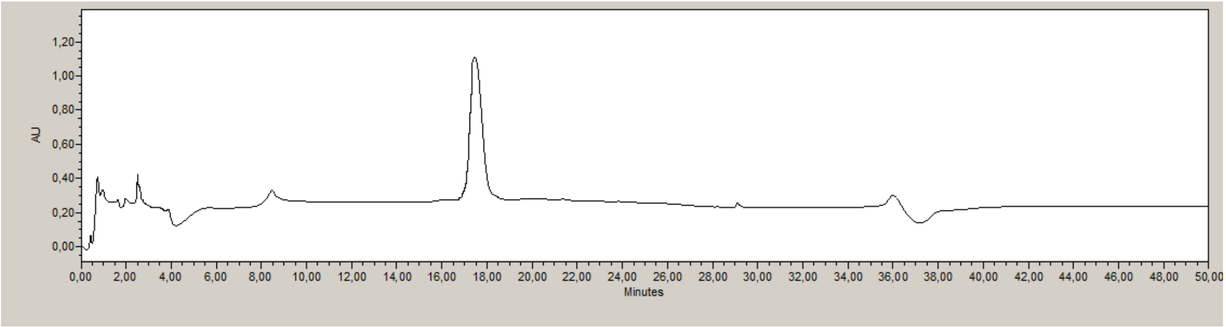

Figure S4. Analytical HPLC chromatogram of peptide Dmt-Tic-c[D-Dap-Phe-Phe-Asp]NH_2 (**4**).

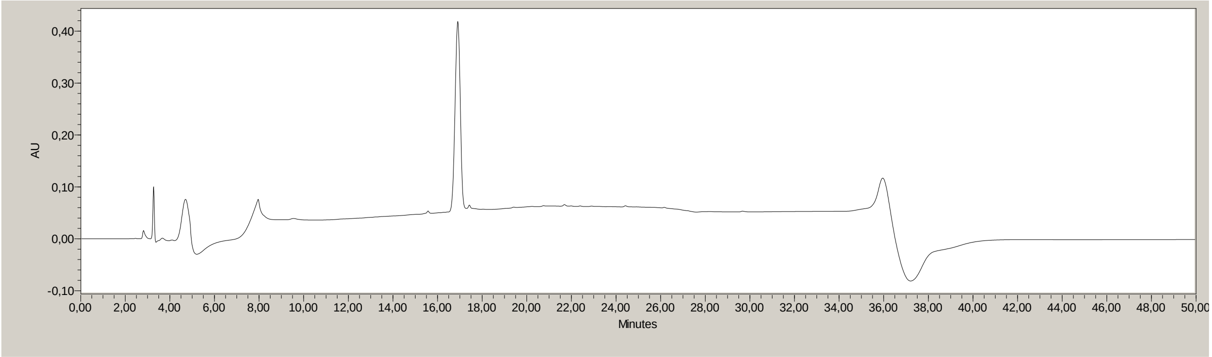

Figure S5 . Analytical HPLC chromatogram of peptide Dmt-Tic-c[D-Lys-Phe-Asp]NH_2 (**5**).

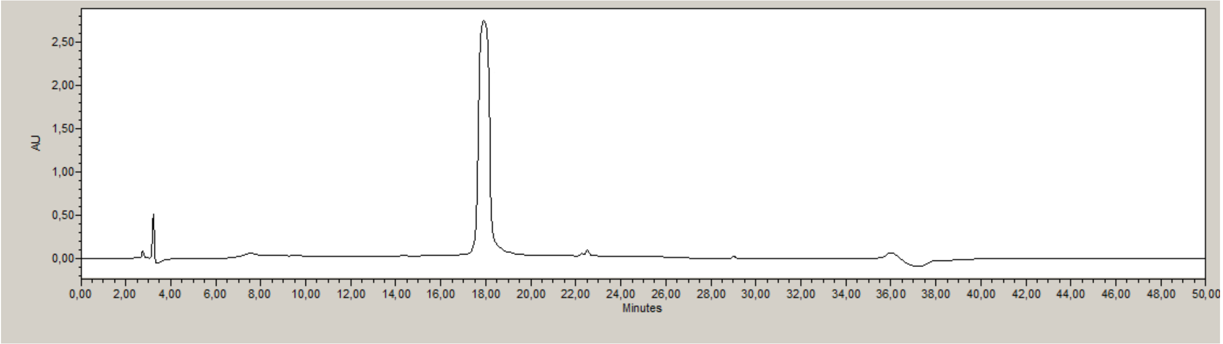

Figure S6. Analytical HPLC chromatogram of peptide Dmt-Tic-c[D-Lys-Phe-Asp]-Tic-Dmt-NH_2 (**6**).

Table S1. Physicochemical data of analogs **1-6**.

No.	Sequence	Formula	m/z $[M + H]^{+a}$		HPLC t_R^b [min]
			Calcd	Obsd	
**1**	Dmt-Tic-c[D-Lys-Phe-Phe-Asp]NH_2	$C_{49}H_{59}N_8O_8$	887.4450	887.4418	17.04
**2**	Dmt-Tic-c[D-Lys-Phe-D-2Nal-Asp]NH_2	$C_{53}H_{61}N_8O_8$	937.4606	**937.4567**	**18.16**
**3**	Dmt-Tic-c[D-Lys-Phe-2,4F_2-Phe-Asp]NH_2	$C_{49}H_{56}F_2N_8O_8$	923.4262	923.4244	17.21
**4**	Dmt-Tic-c[D-Dap-Phe-Phe-Asp]NH_2	$C_{46}H_{52}N_8O_8$	845.3981	845.3997	17.55
**5**	Dmt-Tic-c[D-Lys-Phe-Asp]NH_2	$C_{40}H_{49}N_7O_7$	740.3766	740.3760	16.87

**6**	Dmt-Tic-c[D-Lys-Phe-Asp]-Tic-Dmt-NH$_2$	$C_{61}H_{71}N_9O_{10}$	1090.5396	1090.5345	17.86

[a] Observed by ESI MS$^+$ ionization.

[b] Retention time on a Vydac C$_{18}$ column (4.6 x 250 mm, 5 μm,) using the solvent system of 0.1% TFA in water (A) and 80% acetonitrile in water containing 0.1% TFA (B) and a linear gradient of 0–100% solvent B over 50 min, with a flow rate of 1 mL/min.

High resolution mass spectra were recorded using Shimadzu IT-TOF (ion trap – time-of-flight) mass spectrometer equipped with standard ESI source (Shimadzu, Japan). For CID (collision-induced dissociation) experiments, the singly protonated precursor ions [M+H]$^+$ were selected. The collision energy was adjusted to obtain the optimal fragmentation pattern. Argon was used as a collision gas. The obtained fragments were registered as an MS/MS (tandem mass spectrometry) spectrum.

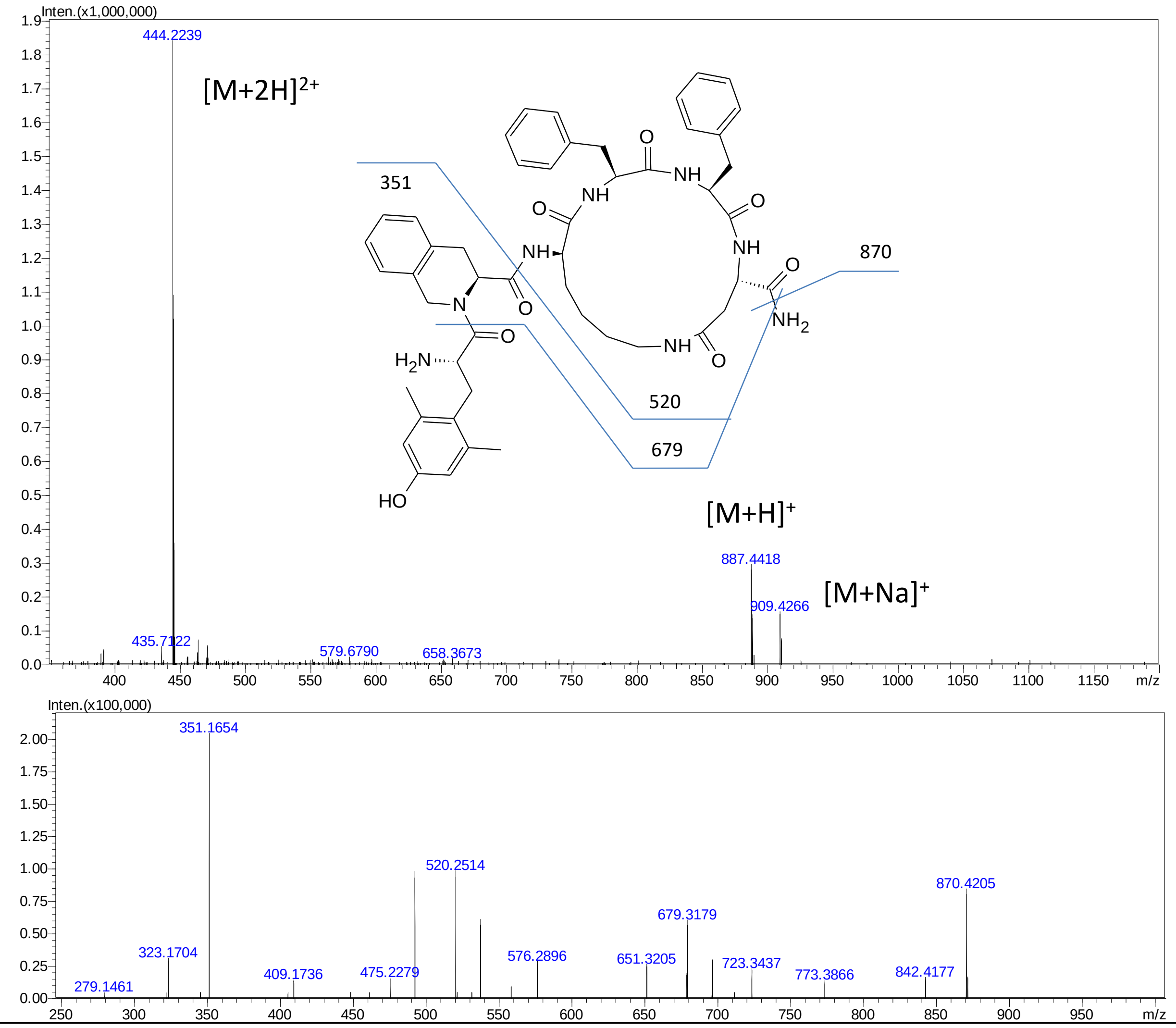

Figure S7. Top panel: High resolution MS spectrum of peptide Dmt-Tic-c[D-Lys-Phe-Phe-Asp]NH_2 (**1**). Bottom panel: High resolution MS/MS spectrum for the [M+H]+ ion. In inset, the fragmentation scheme corresponding to MS/MS spectrum is proposed.

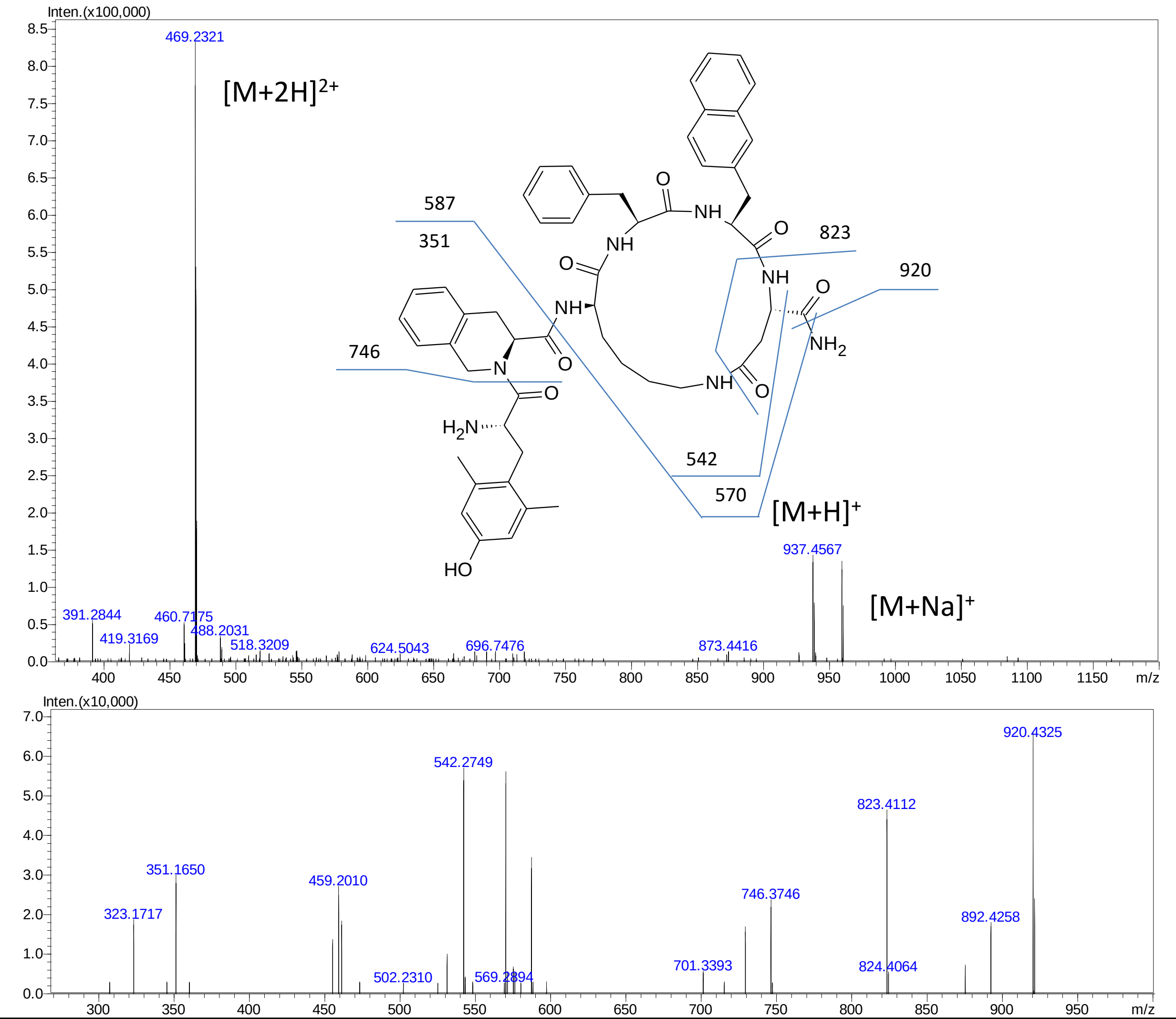

Figure S8. Top panel: High resolution MS spectrum of peptide Dmt-Tic-c[D-Lys-Phe-D-2Nal-Asp]NH_2 (**2**). Bottom panel: High resolution MS/MS spectrum for the [M+H]+ ion. In inset, the fragmentation scheme corresponding to MS/MS spectrum is proposed.

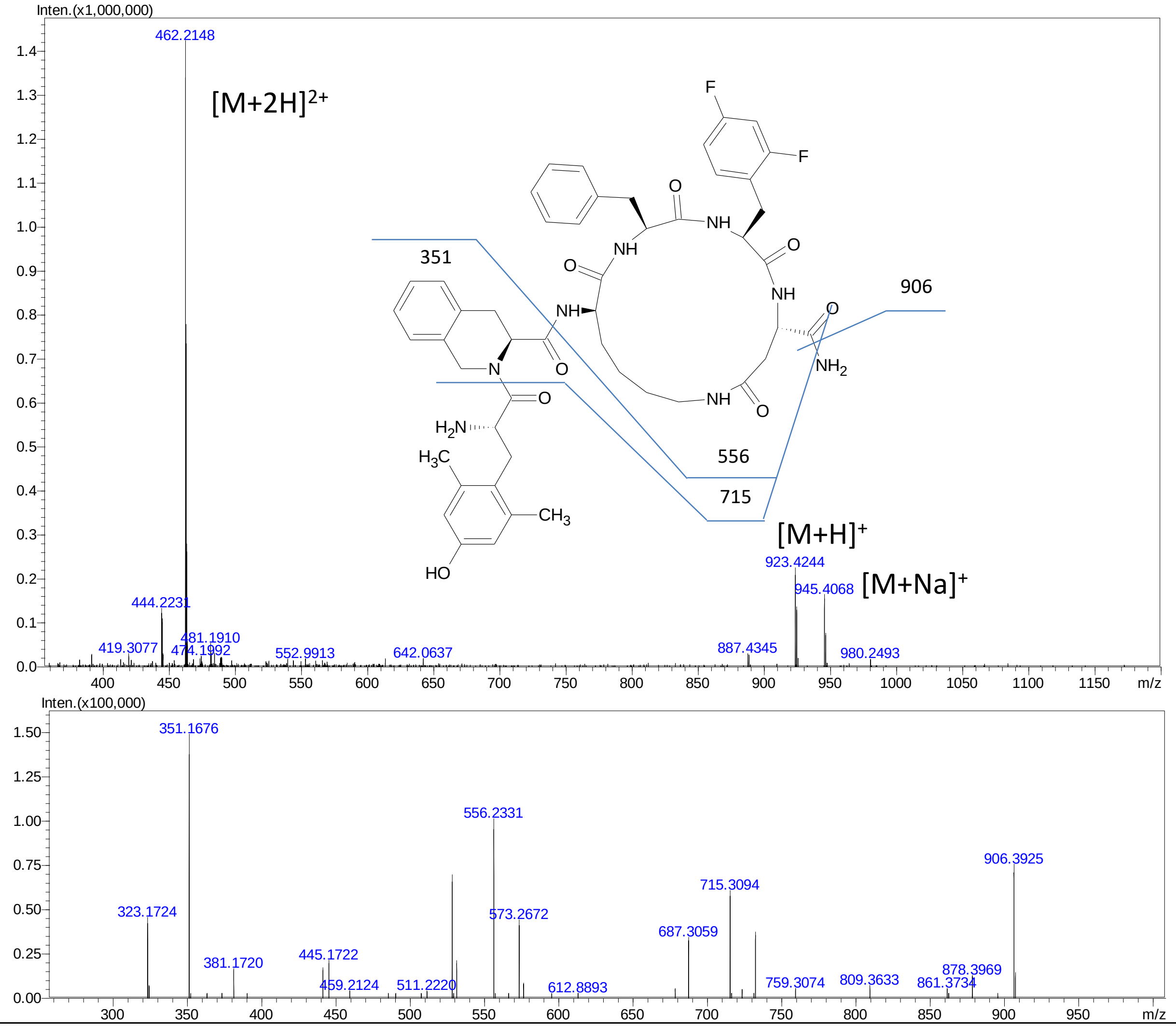

Figure S9. Top panel: High resolution MS spectrum of peptide Dmt-Tic-c[D-Lys-Phe-2,4F$_2$Phe-Asp]NH$_2$ (**3**). Bottom panel: High resolution MS/MS spectrum for the [M+H]+ ion. In inset, the fragmentation scheme corresponding to MS/MS spectrum is proposed.

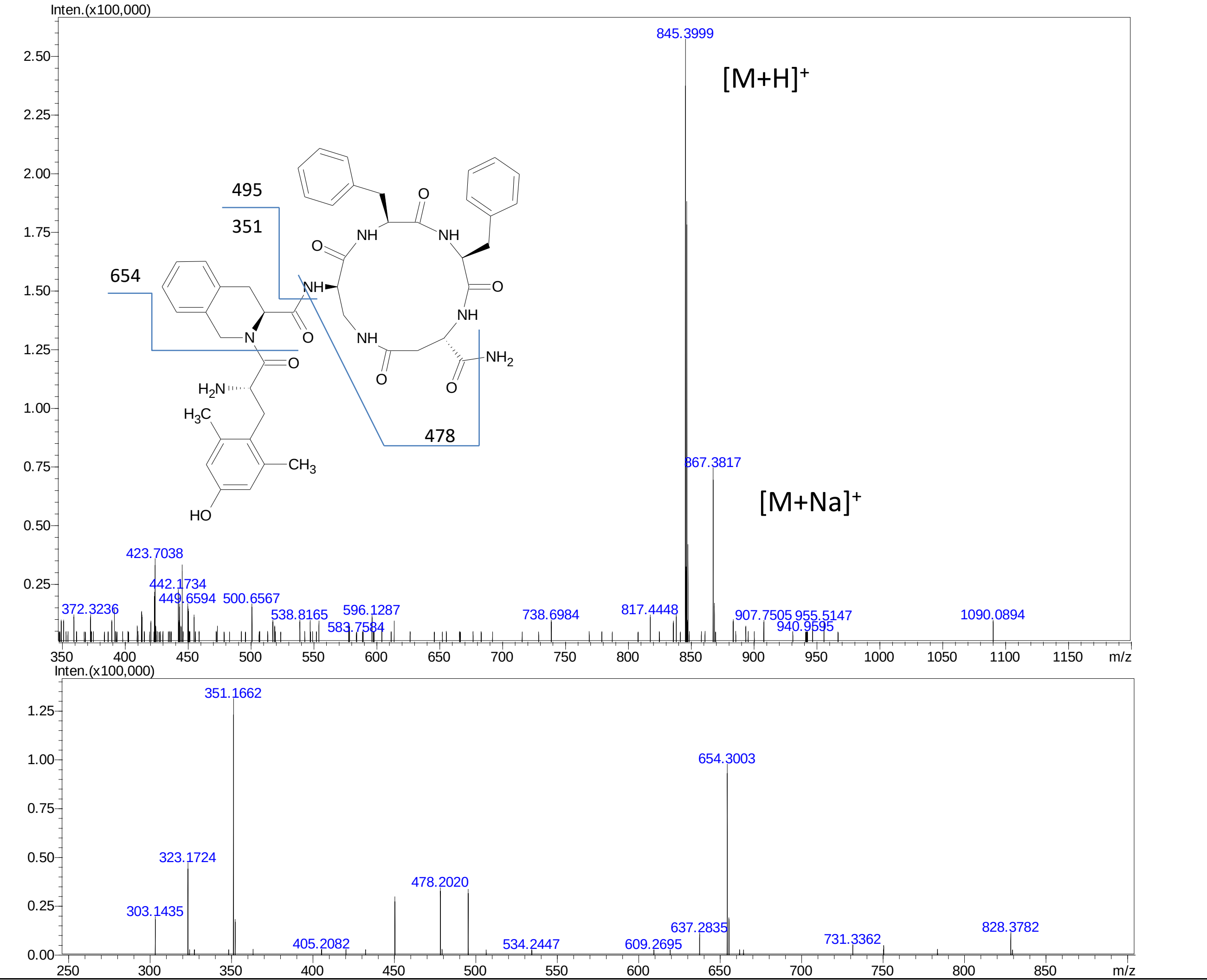

Figure S10. Top panel: High resolution MS spectrum of peptide Dmt-Tic-c[D-Dap-Phe-Phe-Asp]NH$_2$Dmt-(**4**). Bottom panel: High resolution MS/MS spectrum for the [M+H]+ ion. In inset, the fragmentation scheme corresponding to MS/MS spectrum is proposed.

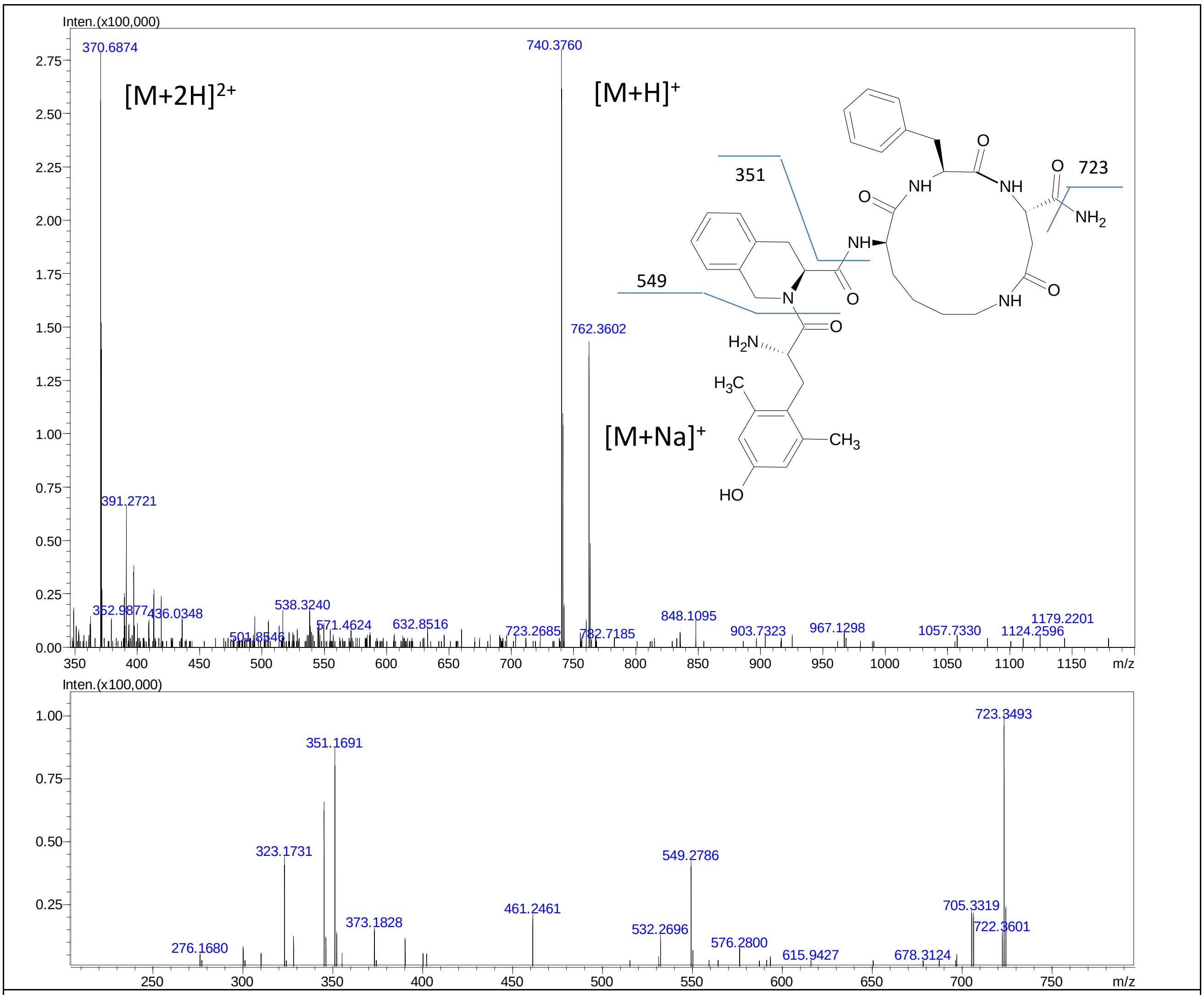

Figure S11. Top panel: High resolution MS spectrum of peptide Dmt-Tic-c[D-Lys-Phe-Asp]NH_2 (**5**). Bottom panel: High resolution MS/MS spectrum for the [M+H]+ ion. In inset, the fragmentation scheme corresponding to MS/MS spectrum is proposed.

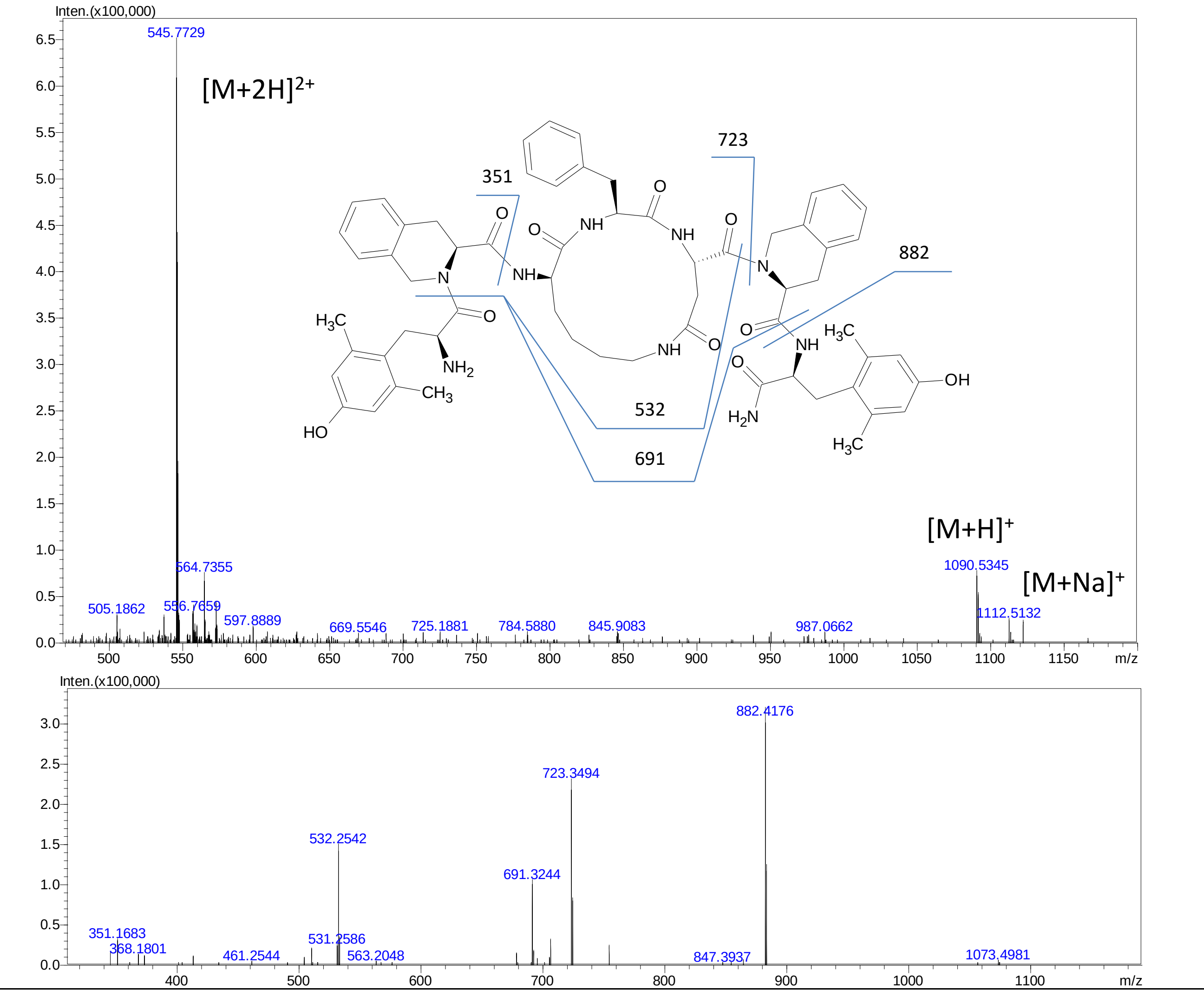

Figure S12. Top panel: High resolution MS spectrum of peptide Dmt-Tic-c[D-Lys-Phe-Asp]-Tic-Dmt-NH$_2$ (**6**). Bottom panel: High resolution MS/MS spectrum for the [M+H]+ ion. In inset, the fragmentation scheme corresponding to MS/MS spectrum is proposed.
